# A Discounted Cash Flow variant to detect the optimal amount of additional burdens in Public-Private Partnership transactions

**DOI:** 10.1016/j.mex.2016.03.003

**Published:** 2016-03-11

**Authors:** Sergio Copiello

**Affiliations:** University IUAV of Venice, Department of Design and Planning, Venice, Italy,

**Keywords:** Discounted cash flow, Discounted Cash Flow, Property Investment Valuation, Public-Private Partnership, Discount rate, Cost of capital, Capital Asset Pricing Model, Risk, Optimization

## Abstract

The Discounted Cash Flow method is a long since well-known tool to assess the feasibility of investment projects, as the background which shapes a broad range of techniques, from the Cost-Benefit Analysis up to the Life-Cycle Cost Analysis. Its rationale lies in the comparison of deferred values, only once they have been discounted back to the present. The DCF variant proposed here fits into a specific application field. It is well-suited to the evaluations required in order to structure equitable transactions under the umbrella of Public-Private Partnership.

• The discount rate relies upon the concept of expected return on equity, instead than on those of weighted average cost of capital, although the latter is the most common reference within the scope of real estate investment valuation.
• Given a feasible project, whose Net Present Value is more than satisfactory, we aim to identify the amount of the additional burdens that could be charged to the project, under the condition of keeping the same economically viable.
• The DCF variant essentially deals with an optimization problem, which can be solved by means of simple one-shot equations, derived from financial mathematics, or through iterative calculations if additional constraints must be considered.

## Graphical abstract

**Figure:**
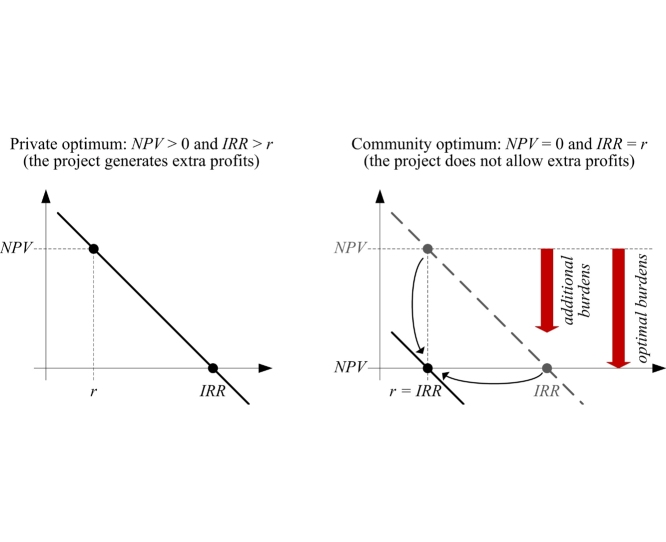

## 1 Method details

### 1.1 Method description

During most of the last century, a variety of techniques and tools aimed at evaluating the feasibility of projects, from several perspectives, have been widely developed. It deserves mention, as the most notable example, the Cost-Benefit Analysis. The literature endorses that its origin lies somewhere back in time, between the second half of the nineteenth and the beginning of the twentieth-century [1], while its practice has widely spread after the Second World War. Furthermore, it has lent itself to be variously modulated, such as towards the cost-revenue analysis, rather than the cost-effectiveness investigation [2]. As another case in point, just bear in mind that the founding of the Life-Cycle Cost Analysis occurred during the mid-seventies. Originally applied within the scope of the US Department of Defense, it was meant to support the choices during the procurement process of weapons [3], [4], especially jet fighters and aircraft carriers [5]. In the meanwhile, its purpose has evolved toward assessing the design, construction and operational features of a variety of goods and services, including manufacturing plants and factory buildings [6]. Moreover, it has been broadly used to compare alternatives with regard to the adoption of energy-efficient measures in the building sector [7], [8].

The aforementioned techniques have a fundamental common characteristic. They adhere to the rationale of the financial mathematics, which consists in the need to discounting back to the present the values expected to occur in the future. Hence, all those analytical tools rely on the Discounted Cash Flow (DCF) method. According to Damodaran’s definition [9], the DCF assumes that “the value of an asset is the present value of the expected cashflows on the asset, discounted back at a rate that reflects the riskiness of these cashflows”. In his survey around valuation approaches [9], the same author identifies the origins of the method in a couple of studies dating back to the first three decades of the past century. The concept of present worth is noticed in Böhm-Bawerk [10] and Marshall [11], who indeed wrote the following sentence about a century ago: “… human nature is so constituted that in estimating the ‘present value’ of a future benefit most people generally make a second deduction from its future value, in the form of what we may call a ‘discount’, that increases with the period for which the benefit is deferred.” Meanwhile, two fundamental studies by Fisher [12], [13] laid the basis for the comparison rules, to which we resort in dealing with multiple alternatives to pick the best one. They are of various kinds but essentially linked to concepts such as those of the present worth and of the rate of return. Just a few years later, Boulding [14] deepened and fully developed the latter of the aforementioned concepts.

During the time span between the mid-sixties and the early seventies, the DCF approach has been progressively adopted in the theory and practice of the real estate appraisal. The literature agrees that several pioneering studies paved the way to the widespread of DCF-based estimates in the branch of property investment valuation. A significant set of these studies can be identified in those published by Downs [15], Dilmore [16] and Ratcliff [17], while a two-part article signed by Greaves [18], originating from his Ph.D. thesis, is widely recognized as the inspiration behind the adoption of DCF by the UK’s appraisers. Just a few years later, the model developed by Marshall [19] marked a further advancement towards the common use of the DCF method in property investment valuations. Such a model exhibits two specific features: explicit projection of future cash flows the former; use of a risk-adjusted discount rate, which involves the concept of opportunity cost of capital, the latter. Due to subsequent enhancements, DCF is now long since a prominent method within the so-called income approach to property appraisal. Moreover, it may be devoted not only to identify the value that can be ascribed to a property, since it is suitable also to support decisions about the feasibility and viability of investing in real estate because its structure reflects the economic fundamentals on which the same investment decisions rely [20].

**Fig. 1:**
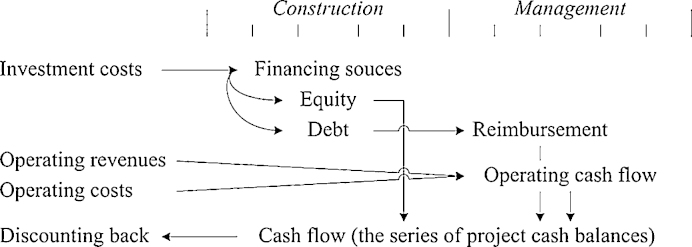
Financial modeling preparatory to a Discounted Cash Flow analysis.

The International Valuation Standards, published by the namesake Council, provide a concise definition of the DCF; it is as follows: “A financial modelling technique based on explicit assumptions regarding the prospective income of a business or property” [21,p. 300]. The development of a DCF analysis entails the need to outline a scheme (Fig. 1) wherein investment costs, operating revenues, and operating costs are projected over the time span under investigation, and processed jointly with other related parameters (Table 1). This leads to identifying the cash flows. Sharing the principle that deferred values are not immediately comparable, the cash flows, in turn, should be discounted back to the present by means of a conversion factor represented by the discount rate. Among the other essentials of DCF, the so-called judgment criteria play a prominent role. The Net Present Value (NPV) expresses the sum of all the ingoing and outgoing cash flows, once they have been discounted at a given discount rate. Therefore, it could be said that the NPV provides a measure of the additional monetary resources generated by implementing the investment. Besides, the Internal Rate of Return (IRR) is a specific discount rate, which has the property to make null the NPV. It represents a relative measure of the profitability of the self-same investment. The equation for the NPV calculation is as follows:

(1) *NPV* = Σ*_i_*_=1..n_*CF_i_*/(1 + *r*)*^n^*

where *CF* is the amount of the cash balances, which in turn overall constitute the cash flow to be discounted at the rate *r*, while *i* represents each time unit of analysis during the time span *n*.

The discount rate estimation is a highly debated issue. The literature points out the prevalence of two schools of thought within the branch of property investment valuation. The first one uses the yields of long-term Treasury bonds as a source [22], relying on the empirical finding that the construction and real estate sectors are generally less risky than other markets. The second approach bases itself on the principle that the discount rate should reflect the combined cost of all the funding sources employed to ensure the covering of investment costs, looking at equity and debt as the main ones; this perspective singles out the concept of Weighted Average Cost of Capital (WACC) [23].

### 1.2 Method remodeling within the scope of public-Private partnership

Before discussing the DCF variant we propose, it appears appropriate to make an introductory digression about the scope of application. Investment projects concerning both private properties and collective infrastructures and facilities are ever more frequently carried out under the umbrella of Public-Private Partnerships (PPPs) [24]. This phenomenon is induced by several circumstances, such as the paucity of public funds intended to provide public goods and services at the urban-wide scale, as well as the will of public bodies to benefit from the entrepreneurial ability of private entities during the production process of the aforementioned goods and services [25].

It has been argued that PPPs lend themselves to drive the decision makers toward investment transactions characterized by a higher degree of social and environmental sustainability [26]. It means that, within the PPP transactions, the private counterparts may be asked to bear additional burdens, in order to bankroll the provision of benefits in behalf of the community. In the matter of the social dimension of sustainability, the literature focuses on the concerns related to the equitable access to the resources [27], as well as to the strengthening of the so-called social capital [28], even through the provision and maintenance of infrastructures and facilities [29]. Besides, the environmental sustainability concerns may relate to the adoption of mitigation measures at the urban-wide scale, as well as to the implementation of solutions aimed to reduce the greenhouse gas emissions at the building scale. A specific branch of action, which ties together the social and environmental aspects, pertains to the provision of energy-efficient affordable dwellings [30], as a way to counteract the phenomena of housing poverty and fuel poverty.

The negotiation process that commonly precedes the signing of a PPP agreement entails the need to be supported by evaluation evidence. The DCF analysis may provide a suitable aid, particularly in order to outline the feasibility degree of a project, as well as to rank the design alternatives according to their economic viability. Nevertheless, to pursue the aforementioned goals, the traditional DCF framework requires being adapted. This article aims to outline a way the DCF may be remodeled, allowing us to organize more equitable PPP transactions.

The DCF variant discussed herein takes advantage of a specific perspective, which can be adopted in order to estimate the discount rate. It strongly differs from the two schools of thought discussed in the previous paragraph, although it still relies on the fundamental concepts of cost of capital and risk. In his 1990’s essay, Kincheloe [31] argues the supremacy of the WACC as a method to estimate the discount rate in the real estate appraisals, so he too belongs to the second of the aforementioned schools of thought. Among the reasons for endorsing this conclusion, the ability of the WACC in representing the capital structure implied in an asset or in a project plays a remarkable role. Especially, the author criticizes the adoption of a discount rate relying on the investor’s expected return on equity. He adversely judges the fact that this alternative method, although correctly applied to a different kind of cash flow, usually leads to underestimating the market value of a property. The previously mentioned study by Ling [23] argued that this last criticism is, at least partly, misplaced. Therefore, we can choose to apply the WACC, as the discount rate, to a certain kind of cash flow, namely the before-debt cash flow from operations. Otherwise, we can opt for the developer’s expected return as the discount rate to be applied to a different kind of cash flow, specifically after the debt reimbursement. In the both cases, we are able to get the same result if the costs of funding sources, which contribute to define the discount rates, are specified correctly over the whole time period of analysis.

Estimating the discount rate by referring to the return expected by the developer on its invested equity carries a remarkable benefit. According to the fundamentals of economic theory, if the financial model (see the previous Fig. 1) explicitly includes all the pertinent costs, namely the remuneration owed to all the employed factors of production, then the NPV represents a kind of extra profit. Indeed, such factors include also the entrepreneurship and the involved properties − which can be fairly remunerated by dedicated cost items − as well as the financial capital − whose expected remuneration is expressed precisely by the discount rate. This awareness opens the doors to pursue our original goal, that is to say, limiting the extra profit by imposing additional burdens on the private entities involved in PPP transactions, so they may become more equitable.

**Fig. 2:**
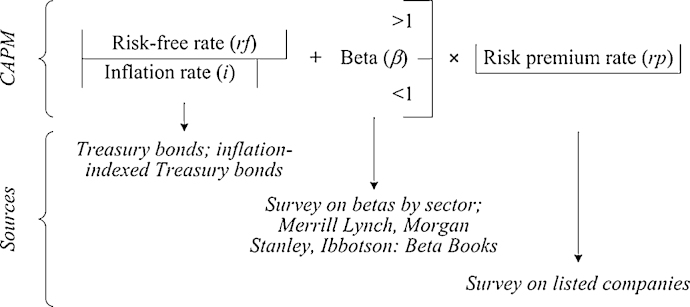
Structure of the discount rate and estimating sources.

Consistently with the above-described conceptual framework, within the customization we propose here, the discount rate estimation relies on the Capital Asset Pricing Model (CAPM). The rationale of the CAPM is summarized in its three basic terms: a market risk premium rate, additional to a base rate, allowing to express the return achievable from somehow risk-free investments, and a beta parameter, which specifically takes account of the not diversifiable risk [32]. The standard equation is as follows:

(2) *k_e_* = *rf* + β·*rp*

where *k_e_* is the discount rate estimated as the expected return on equity, *rf* stands for the rate of return of the most risk-free investments, *rp* is the market risk premium rate and β is a parameter varying around unity, which represents the degree of exposure to the average market risk. The literature long since has discussed a broad range of data sources for gathering information in order to estimate the terms constituting the CAPM. Moreover, several studies provide their own estimates too. As an example, you can see the appendices in Damodaran [33]. The structure of the discount rate, according to Eq. (2), and some exemplifying estimating sources are further shown in Fig. 2.

**Fig. 3:**
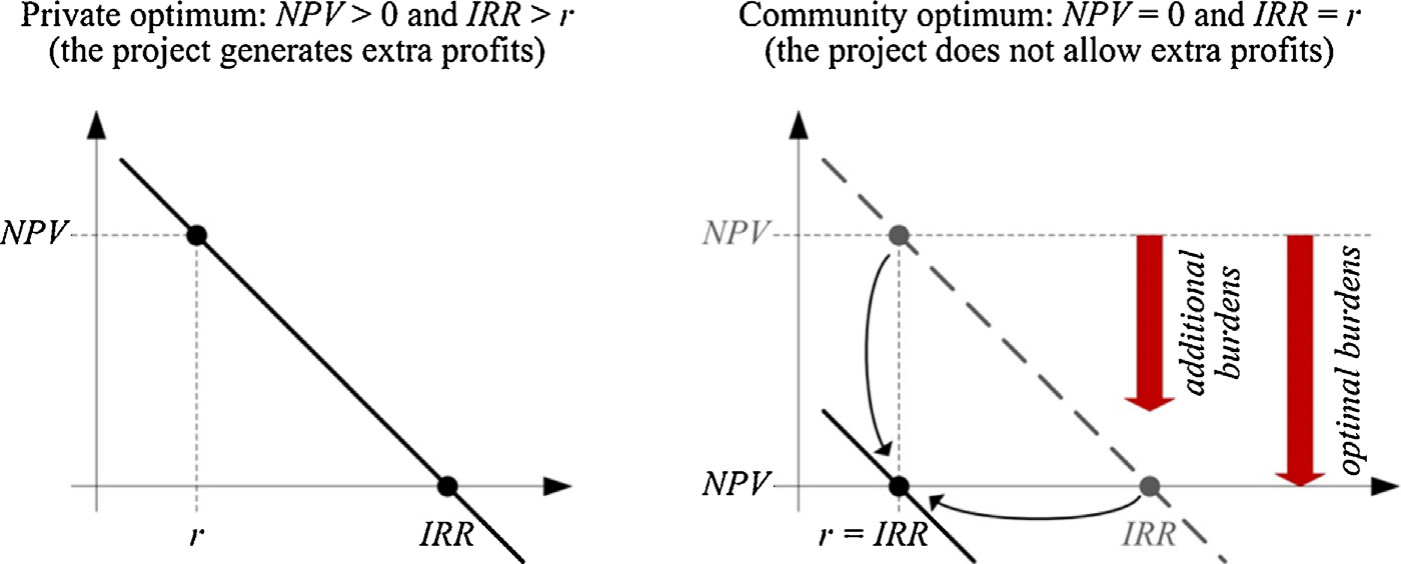
Role of additional burdens within the relationship between discount rate, NPV, and IRR.

Once known the values of NPV and IRR for the project under evaluation, the DCF variant entails essentially an optimization problem, which can be solved through simple one-shot equations, or else by iterative calculations if additional constraints must be considered. Since the costs usually precede the revenues, the NPV turns out to be a decreasing function of the discount rate. Let us assume to face an economically viable project; therefore, for a given discount rate, we achieve a positive NPV and an IRR higher than the same discount rate (Fig. 3, on the left side). Additional burdens, which lower the NPV and the IRR, may be charged to the project developer, until the self-same NPV comes close to zero (Fig. 3, on the right side). An IRR exactly equal to the expected return on equity, namely to the discount rate *k_e_*, represents the limit condition to be satisfied in order to judge the project still economically viable. If such additional burdens are foreseen in the form of a lump-sum payment *a*, which will occur at time *t*, as is the case of public works built by the developer in behalf of the community, their calculation is as follows:

(3) *a* = *NPV*·(1 + *k_e_*)*^t^*

which represents just a mere deferral of the NPV amount. Otherwise, if the additional burdens are foreseen as a periodic payment, as is the case of a concession fee *c* to be paid on a yearly basis from time *t* to time *n*, the calculation is as follows:

(4) *c* = *NPV*·(1 + *k_e_*)*^t−1^*·[*k_e_*·(1 + *k_e_*)*^n^*^−*t*^]/[(1 + *k_e_*)*^n^*^−*t*^ − 1]

### 1.3 Method validation

**Fig. 4:**
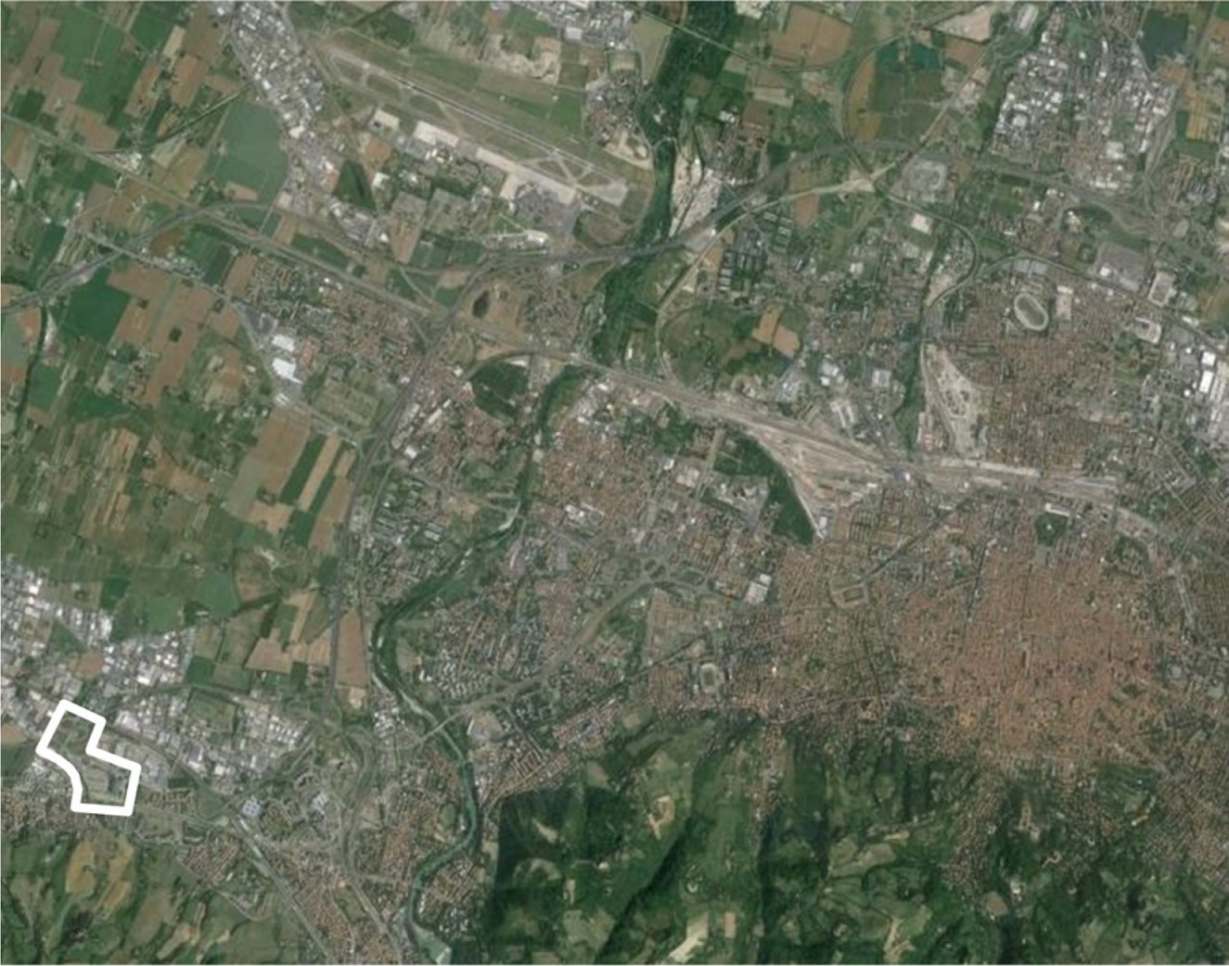
Location of the case study, southwest of the city of Bologna.

**Fig. 5:**
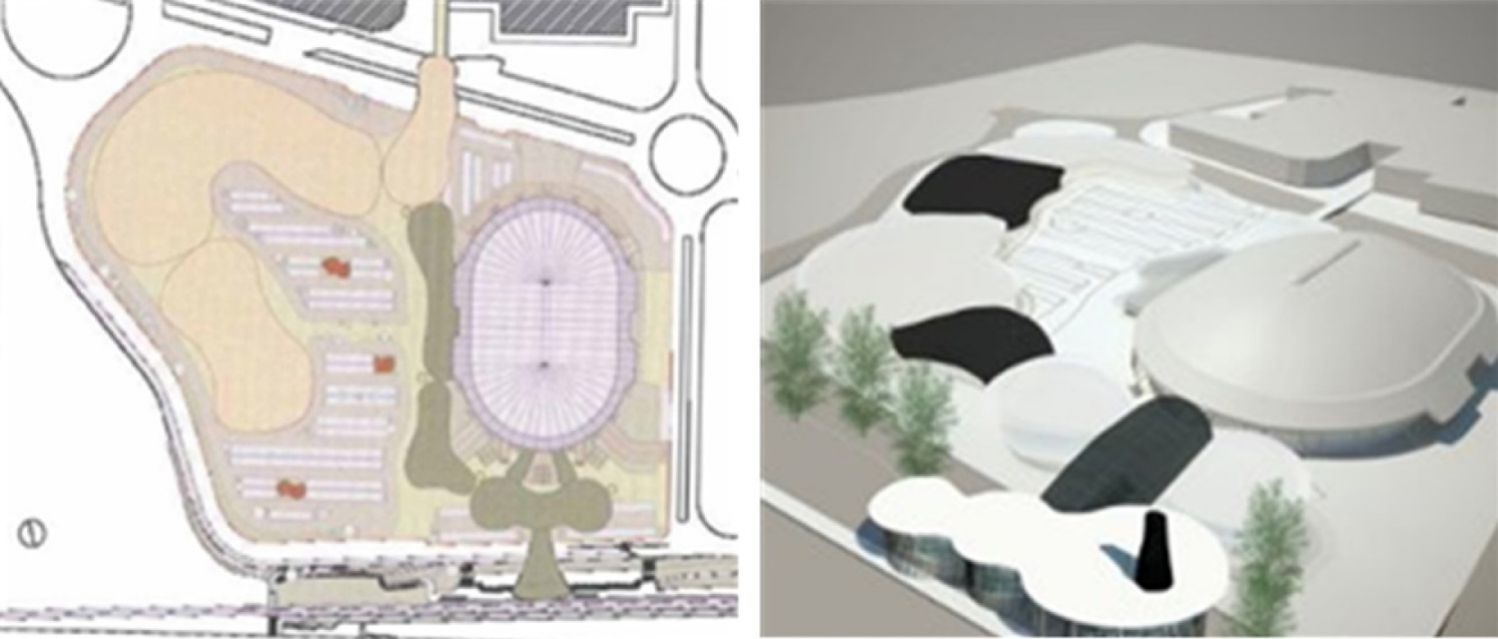
Case study: planning and design (source: adapted from the developers’ master plan).

The DCF variant outlined here has been tested and validated through a case study application. The case consists of the rehabilitation of a sports complex located in the suburbs of Bologna (Fig. 4, Fig. 5), a medium-sized city in Northern Italy. The case perfectly fits the circumstances for which the DCF variant has been proposed: it is an urban renewal project that implies the need to incur property investments, to be carried out under the umbrella of a Public-Private Partnership. Here is the case history.

During the early nineties, a new indoor sports arena mainly meant to host basketball matches was built on the southwestern outskirts of Bologna. Its occasional and desultory usage led to a sudden physical and functional decay. In 2008, a private company came forward to renew the arena and the surrounding area. It submitted a project to the Municipality, in order to be awarded the right to carry out the works, according to a long-term ground lease, which is referred to as surface right in the national legal system. The ground lease was foreseen to last up to ninety-nine years. The project contemplated the rehabilitation of the building, in order to make it suitable to host other events beyond the sports competitions, such as concerts, exhibitions, and corporate conventions. Moreover, the feasibility of the renewal project was pursued by adding a new shopping mall.

The subsequent negotiation process among the involved public bodies, namely Municipal and Provincial authorities, and the private developer was intended to point out the transaction characteristics: the site management conditions, according to the entrepreneurial ability of the private entity, on the one hand; the community benefits to be charged to the same private entity, in the form of a yearly concession fee, on the other hand. Therefore, during the second half of 2010, the negotiation process was supported by the previously defined DCF variant.

An appraisal of the fundamental financial and macroeconomic parameters was carried out prior to developing the cash flows, according to the scheme drafted in the previous Fig. 2. Information and data were gathered from the sources there mentioned, as they are summarized in Table 2. The risk-free rate was estimated looking to the average yield offered by the long-term Treasury bonds, with a maturity of 30 years (the longest available in Italy), which were placed to the investors through auctions during the time span from 2005 to 2010. Both the nominal rate (*rf_n_*) and the real rate (*rf_r_*) were taken into account, the latter by referring to the inflation-indexed Treasury bonds. Hence, the inflation rate (*i*) was extrapolated according to the following calculation:

(5) *i* = (1 + *rf_n_*)/(1 + *rf_r_*) − 1

The choice of the beta parameter (β) was suggested by the data disseminated by Damodaran (see http://pages.stern.nyu.edu/∼adamodar/). Accordingly, on the whole, the project was considered more prone to risk than the average. Finally, the risk premium rate (*rp*) was inferred from the data included in several periodic reports, released by survey institutions and focused on the companies listed on the Milan stock exchange. The listed companies taken into account were those operating in fields akin to the project, namely construction sector, real estate, utilities, leisure and entertainment industry. Instead than applying the inflation rate to the operating costs and revenues, we opted for the use of a real (deflated) discount rate. The outcome was a 6.77% expected return on equity, net of the inflation.

**Fig. 6:**
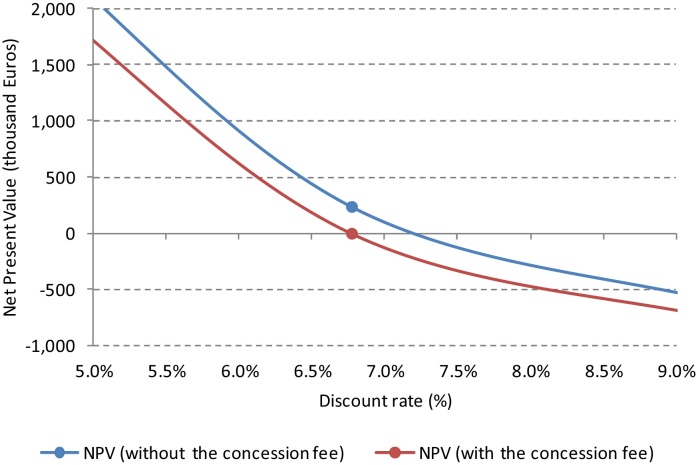
Comparison between the NPVs with and without the concession fee.

The Table 3, Table 4, Table 5 show the cash flow for the first couple of decades in the time span under analysis, while for the complete cash flow please refer to the supplementary materials here enclosed. The results were fairly good since the NPV turned out to be positive (about 238 thousand Euros). According to the previous Eq. (4), this allowed to estimate the maximum yearly amount of the concession fee (about 26 thousand Euros), to be paid from the beginning of the management after the completion of the refurbishment works (year 8), until the expiration date of the ground lease (year 99). As may be observed in the complete cash flow, and as further shown in Fig. 6, the concession fee represents exactly the amount of resources which lowers the NPV until it becomes null.

## Figures and Tables

**Table 1:** Parameters involved in the financial model.

*Technical and operating parameters*	*Tax parameters*	*Financial parameters*	*Macroeconomic parameters*
Investment costs (funding sources)	Amortization and taxation (tax base and rates)	Cost of equity and cost of debt (interest rates)	–
Operating revenues		–	Inflation rate
Operating costs		–	

**Table 2:** Estimates of financial and macroeconomic parameters.

*Parameter*		*Value*	*Estimating source*
Nominal risk-free rate	*rf_n_*	4.87%	Long-term Treasury bond (maturity of 30 years, average rate from the year 2005 to 2010)
Real risk-free rate	*rf_r_*	2.37%	Inflation-indexed Treasury bond (maturity of 30 years, average rate from the year 2005 to 2010)
Inflation rate	*i* = (1 + *rf_n_*)/(1 + *rf_r_*) − 1	2.44%	
Beta	*β*	1.1	Betas by sector calculated by Damodaran (see http://pages.stern.nyu.edu/∼adamodar/)
Risk premium rate	*rp*	4.00%	Periodic reports, released by survey institutes, about listed companies on the Milan stock exchange
Expected return on equity	*ke* = *rfr* + (β·*rp*)	6.77%	

**Table 3:** Cash flow, values in thousand Euros, results are rounded to thousands.

*Time*	*1*	*2*	*3*	*4*	*5*	*6*	*7*	*8*	*9*
Investment costs									
Equity conferral (a)	−698	−1	0	−11	−442	−795	−819	0	0
Net operating revenues (b)									
Sports arena	0	232	232	232	232	232	232	495	495
Shopping mall	0	0	0	0	0	0	0	564	564
Financial and tax items									
Debt reimbursement (c)	0	−207	−207	−207	−207	−207	−207	−875	−875
Taxes	0	0	0	0	0	0	0	−35	−35
Discounting back									
Cash flow	−698	24	25	14	−417	−770	−794	149	149
Discount rate	6.77%	6.77%	6.77%	6.77%	6.77%	6.77%	6.77%	6.77%	6.77%
Discount factor	0.937	0.877	0.822	0.769	0.721	0.675	0.632	0.592	0.555
Discounted cash flow	−654	21	21	11	−301	−520	−502	88	83

(a) Equity is intended to finance construction, refurbishment, and design costs.

(b) Operating costs are already deducted from gross operating revenues.

(c) Debt is intended to finance part of the investment costs and the overall maintenance expenses.

**Table 4:** Cash flow, values in thousand Euros, results are rounded to thousands.

*Time*	*10*	*11*	*12*	*13*	*14*	*15*	*16*	*17*	*18*
Investment costs									
Equity conferral	0	0	0	0	0	0	0	0	0
Net operating revenues									
Sports arena	495	495	495	495	495	495	495	495	495
Shopping mall	564	564	564	564	564	564	564	564	564
Financial and tax items									
Debt reimbursement	−875	−875	−875	−875	−875	−875	−875	−875	−875
Taxes	−35	−35	−35	−35	−35	−35	−35	−35	−35
Discounting back									
Cash flow	149	149	149	149	149	149	149	149	149
Discount rate	6.77%	6.77%	6.77%	6.77%	6.77%	6.77%	6.77%	6.77%	6.77%
Discount factor	0.519	0.486	0.456	0.427	0.400	0.374	0.351	0.328	0.308
Discounted cash flow	77	72	68	64	60	56	52	49	46

**Table 5:** Cash flow, values in thousand Euros, results are rounded to thousands.

*Time*	*19*	*20*	*21*	*22*	*23*	*24*	*25*	*26*	*27*
Investment costs									
Equity conferral	0	0	0	0	0	0	0	0	0
Net operating revenues									
Sports arena	495	495	495	495	495	495	495	495	495
Shopping mall	564	564	564	564	564	564	564	564	564
Financial and tax items									
Debt reimbursement	−875	−875	−875	−875	−875	−875	−752	−752	−752
Taxes	−39	−46	−53	−82	−94	−97	−174	−194	−195
Discounting back									
Cash flow	145	138	131	102	90	87	133	113	112
Discount rate	6.77%	6.77%	6.77%	6.77%	6.77%	6.77%	6.77%	6.77%	6.77%
Discount factor	0.288	0.270	0.253	0.237	0.222	0.208	0.194	0.182	0.171
Discounted cash flow	42	37	33	24	20	18	26	21	19
